# Topical Administration of Bosentan Prevents Retinal Neurodegeneration in Experimental Diabetes

**DOI:** 10.3390/ijms19113578

**Published:** 2018-11-13

**Authors:** Patricia Bogdanov, Olga Simó-Servat, Joel Sampedro, Cristina Solà-Adell, Marta Garcia-Ramírez, Hugo Ramos, Marta Guerrero, Josep Maria Suñé-Negre, Josep Ramon Ticó, Bruno Montoro, Vicente Durán, Luís Arias, Cristina Hernández, Rafael Simó

**Affiliations:** 1 Diabetes and Metabolism Research Unit, Vall d’Hebron Research Institute, Universitat Autònoma de Barcelona, 08035 Barcelona, Spain; patricia.bogdanov@vhir.org (P.B.); olga.simo@vhir.org (O.S.-S.); joel.sampedro@vhir.org (J.S.); cristina.sola@vhir.org (C.S.-A.); marta.garcia.ramirez@vhir.org (M.G.-R); hugo.ramos@vhir.org (H.R.); cristina.hernandez@vhir.org (C.H.); 2 Centro de Investigación Biomédica en Red de Diabetes y Enfermedades Metabólicas Asociadas (CIBERDEM), Instituto de Salud Carlos III (ISCIII), 28029 Madrid, Spain; 3 Medical Mix S.L.U., 08174 San Cugat del VallèsBarcelona, Spain; mguerrero@medicalmix.com (M.G.); vduran@medicalmix.com (V.D.); 4 Department of Pharmacy and Pharmaceutical Technology and Physical Chemistry, University of Barcelona, 08028 Barcelona, Spain; jmsune@ub.edu (J.M.S.-N.); jrtico@ub.edu (J.R.T.); 5 Pharmacology Department, Vall d’Hebron Hospital, 08035 Barcelona, Spain; bmontoro@vhebron.net; 6 Department of Ophthalmology, Bellvitge University Hospital, University of Barcelona, 08907 Hospitalet del LLobregat, Barcelona, Spain; luisariasbarquet@gmail.com

**Keywords:** diabetic retinopathy, retinal neurodegeneration, endothelin-1, bosentan, db/db mouse

## Abstract

Experimental evidence suggests that endothelin 1 (ET-1) is involved in the development of retinal microvascular abnormalities induced by diabetes. The effects of ET-1 are mediated by endothelin A- and B-receptors (ETA and ETB). Endothelin B-receptors activation mediates retinal neurodegeneration but there are no data regarding the effectiveness of ETB receptor blockage in arresting retinal neurodegeneration induced by diabetes. The main aim of the present study was to assess the usefulness of topical administration of bosentan (a dual endothelin receptor antagonist) in preventing retinal neurodegeneration in diabetic (db/db) mice. For this purpose, db/db mice aged 10 weeks were treated with one drop of bosentan (5 mg/mL, *n* = 6) or vehicle (*n* = 6) administered twice daily for 14 days. Six non-diabetic (db/+) mice matched by age were included as the control group. Glial activation was evaluated by immunofluorescence using specific antibodies against glial fibrillary acidic protein (GFAP). Apoptosis was assessed by TUNEL method. A pharmacokinetic study was performed in rabbits. We found that topical administration of bosentan resulted in a significant decrease of reactive gliosis and apoptosis. The results of the pharmacokinetic study suggested that bosentan reached the retina through the trans-scleral route. We conclude that topical administration of bosentan was effective in preventing neurodegeneration in the diabetic retina and, therefore, could be a good candidate to be tested in clinical trials.

## 1. Introduction

Diabetic retinopathy (DR) is the most frequent complication of diabetes and it is the leading cause of preventable blindness among the working-age population in developed countries [1]. Current treatments for DR such as laser photocoagulation, intravitreous injections of corticosteroids or anti-vascular endothelial growth factor (VEGF) agents are indicated in too-advanced stages of the disease and are associated with significant adverse effects [2]. Therefore, new pharmacological treatments for the early stages of the disease are needed. 

The concept of DR as a microvascular disease has evolved into that of a more complex diabetic complication in which neurodegeneration plays a significant role [3,4,5]. In fact, the American Diabetes Association has recently defined DR as a highly specific neurovascular complication involving progressive disruption of the inter-dependence between multiple cell-types in the retina [6]. The neurovascular unit (NVU) is composed of different types of cells and its impairment is a primary event in the pathogenesis of DR [4,5]. The mechanisms underlying neurovascular coupling are complex, and the upregulation of endothelin-1 (ET-1) detected in the diabetic retina could participate in both early microvascular impairment and neurodegeneration [5].

Results from several studies in streptozotocin-induced diabetic rats have suggested a role of ET-1 in the pathogenesis of microvascular abnormalities in DR [7]. Furthermore, ET-1 antagonists were found to prevent some of the underlying mechanisms of microangiopathic disease in the retina in diabetic animal models [8,9,10,11]. Two receptor subtypes, endothelin A- and B-receptors (ETA and ETB), mediate the effects of ET-1 [12]. In recent years the deleterious role of ETB activation in terms of neurodegeneration has been demonstrated in several experimental models [13,14,15] but there is no information regarding the potential beneficial effects of blocking ETB receptors in DR. Apart from ET-1, there is some evidence that ET-1 receptors are also increased in the retina of type 1 [16,17,18] and type 2 [19] diabetic experimental models but these interesting observations need further confirmation. Furthermore, ET-1 and its receptors are expressed in human retinas [20], but to the best of our knowledge whether this is also true in the very early stages of DR remains to be elucidated. 

When the early stages of DR are the therapeutic target, it would be inconceivable to recommend an aggressive treatment such as intravitreal injections. On the other hand, systemic administration of drugs blocking ET-1 could hardly reach the retina at pharmacological concentrations and, in addition, could have serious adverse effects. The use of eye drops has not been considered an appropriate route for the administration of drugs aimed at preventing or arresting DR because of the general assumption that they do not reach the retina. However, there is emerging evidence to show that many drugs are able to reach the retina in pharmacological concentrations, at least in animal models [2].

On this basis, the aims of the present study were: (1) To compare the expression of ET-1 and its receptors (ETA and ETB) in the retina between diabetic and non-diabetic humans and mice; (2) To assess the usefulness of ocular topical administration of bosentan (a dual endothelin receptor antagonist) in preventing retinal neurodegeneration, an early event in the pathogenesis of DR in diabetic mice; (3) To investigate in vitro the effect of bosentan on TNF-α-induced hyperpermeability and on the upregulation of VEGF induced by the diabetic milieu. 

## 2. Results

### 2.1. Endothelin-1 And Its Receptors A (ETA) And B (ETB) Were Upregulated in the Retina of Diabetic Donors

**Figure 1:**
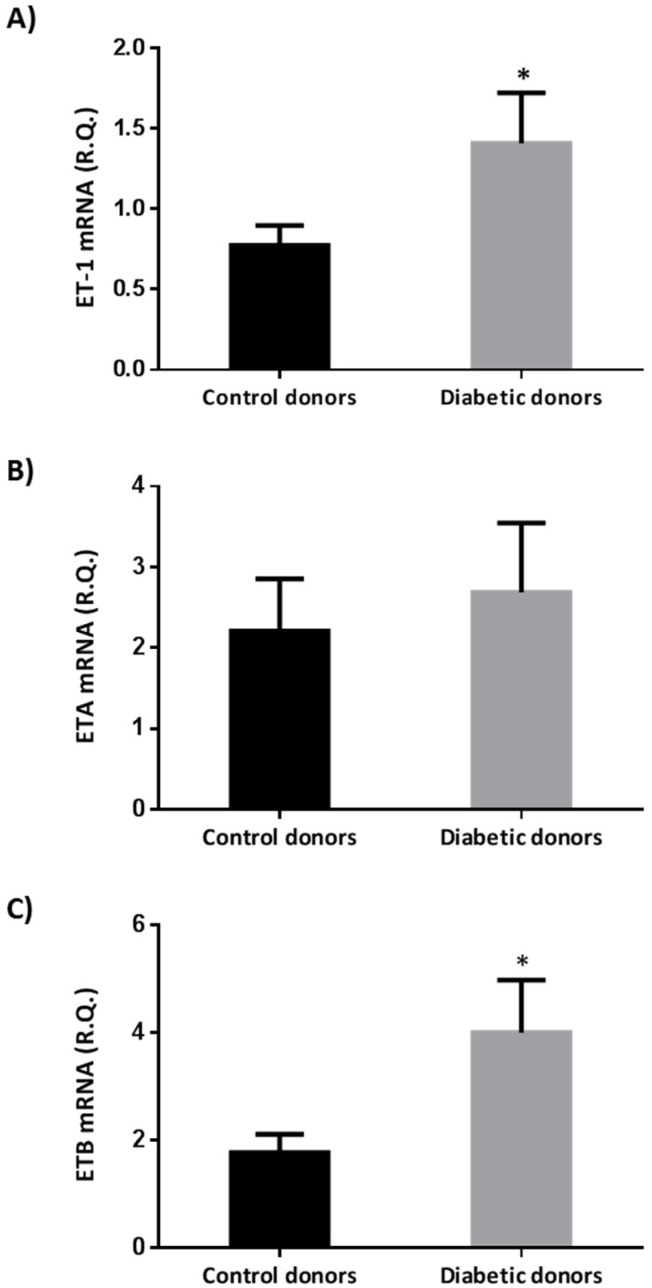
Real-time quantitative RT-PCR analysis of Endothelin-1 (ET-1) mRNA (**A**) endothelin A-receptors (ETA) mRNA (**B**), and endothelin B-receptors (ETB) mRNA (**C**) in human retinas from diabetic and non-diabetic donors. The study was performed in 12 donors with diabetes and 12 donors without diabetes. R.Q.: Relative quantification. Data are mean ± standard error. The Student *t* test was used for comparisons. * *p* < 0.05.

We found that ET-1 and ETB were upregulated in retinas from diabetic donors in comparison with retinas from non-diabetic donors (Figure 1). Endothelin A-receptors were also upregulated in the retinas from diabetic donors, but their increase did not achieve statistical significance.

### 2.2. Bosentan Ameliorated the Upregulation of Endothelin-1 And Its Receptors A (ETA) And B (ETB) in the Retinas of Diabetic Mice

**Figure 2:**
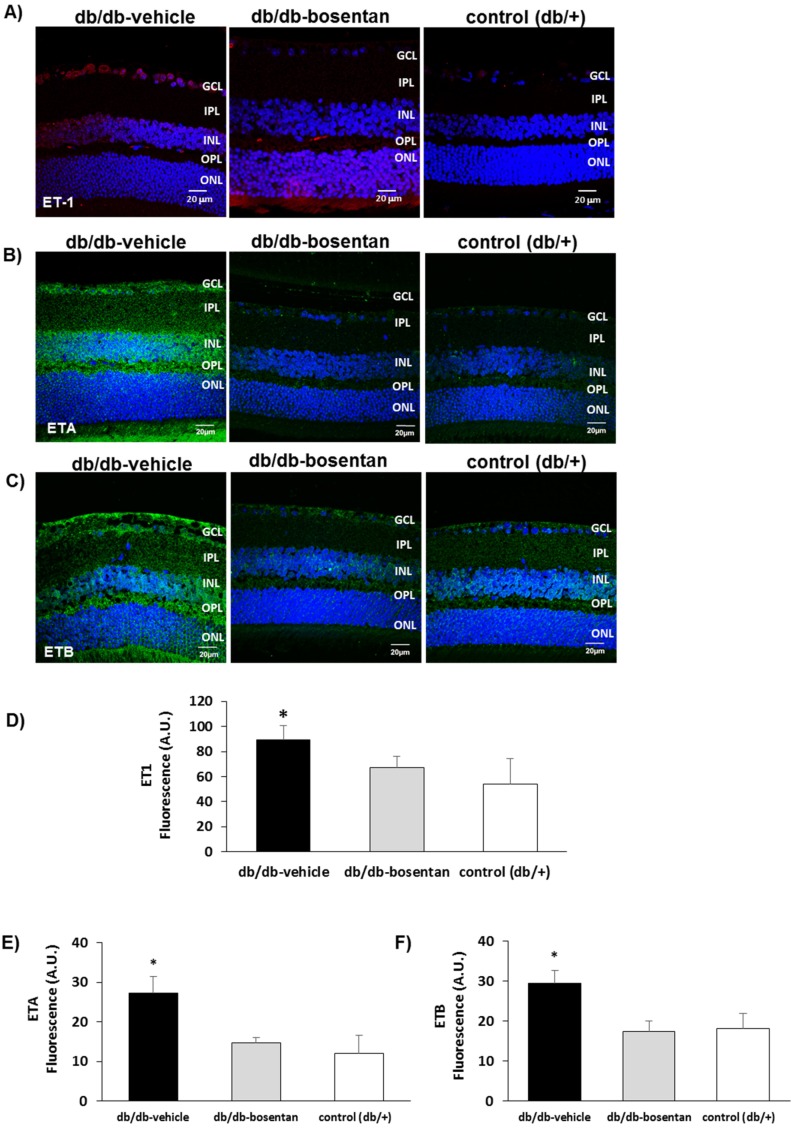
(**A**) Comparison of endothelin-1 immunofluorescence (red) between representative samples from a diabetic mouse (db/db) mouse treated with vehicle, a diabetic mouse treated with bosentan, and a non-diabetic mouse (db/+); (**B**) Comparison of endothelin receptor A immunofluorescence (green) between representative samples from a diabetic mouse (db/db) mouse treated with vehicle, a diabetic mouse treated with bosentan, and a non-diabetic mouse (db/+); (**C**) Comparison of endothelin receptor B immunofluorescence (green) between representative samples from a diabetic mouse (db/db) treated with vehicle, a diabetic mouse treated with bosentan, and a non-diabetic mouse (db/+). GCL: Ganglion cell layer; IPL: Inner plexiform layer; INL: Inner cell layer; OPL: Outer plexiform layer; ONL: Outer nuclear layer. Scale bar: 20 µm; (**D**–**F**) Quantification of immunofluorescence. AU: Arbitrary units. Data are expressed as mean ± SD. * *p* < 0.05 in comparison with the other groups.

The expression of ET-1 was significantly higher in diabetic mice (db/db) in comparison with non-diabetic mice (Figure 2). In addition, the content of both ETA and ETB were significantly higher in diabetic (db/db) mice than in non-diabetic control mice (Figure 2). Bosentan was able to significantly reduce the upregulation of ET-1, ETA and ETB induced by diabetes.

### 2.3. Bosentan Prevented Diabetes-Induced Neurodegeneration in Diabetic Mice

Blood glucose concentration and body weight at the end of treatment were similar in db/db mice treated with bosentan than in db/db mice treated with vehicle.

### 2.4. Glial Activation

**Figure 3:**
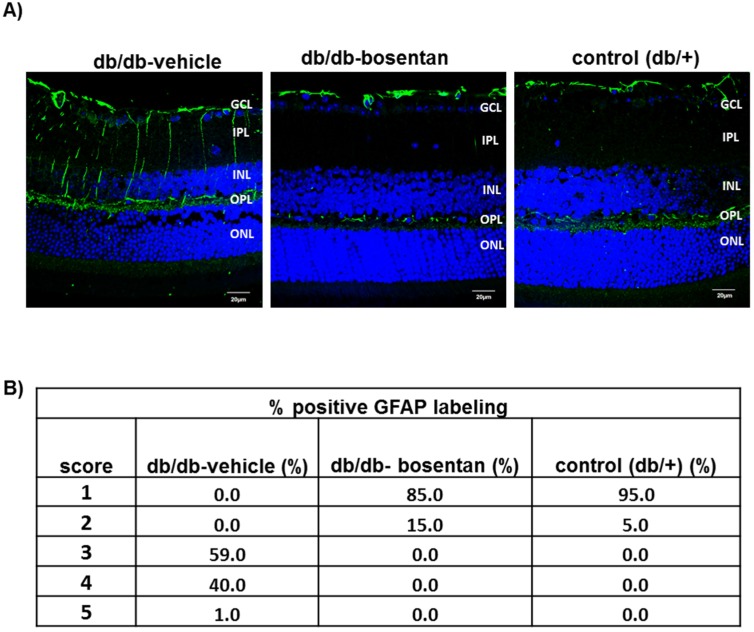
Glial activation. (**A**) Comparison of glial fibrillary acidic protein (GFAP) immunofluorescence (green) between representative samples from a db/db mouse treated with vehicle, a db/db mouse treated with bosentan and a non-diabetic mouse. In the diabetic mouse treated with vehicle, the Müller cells’ endfeet show abundant GFAP immunofluorescence and the radial processes stain intensely throughout both the inner and outer retina. Nuclei were labeled with DAPI (blue). GCL: Ganglion cell layer; IPL: Inner plexiform layer; INL: Inner cell layer; OPL: Outer plexiform layer; ONL: Outer nuclear layer; (**B**) Quantification of glial activation based on extent of GFAP staining. *n =* 6 mice per group (10 sections per retina).

Glial fibrillary acidic protein expression was confined to the retinal ganglion cell layer in non-diabetic mice and therefore the GFAP score was ≤2 (Figure 3A,B). The diabetic mice treated with vehicle presented significant higher GFAP expression than non-diabetic mice matched by age. Thus, 100% of diabetic mice presented a GFAP score ≥3. Bosentan administration for two weeks resulted in a significant decrease of reactive gliosis, and the GFAP score of the mice treated with bosentan was <3 in all cases (Figure 3A,B).

### 2.5. Retinal Apoptosis

**Figure 4:**
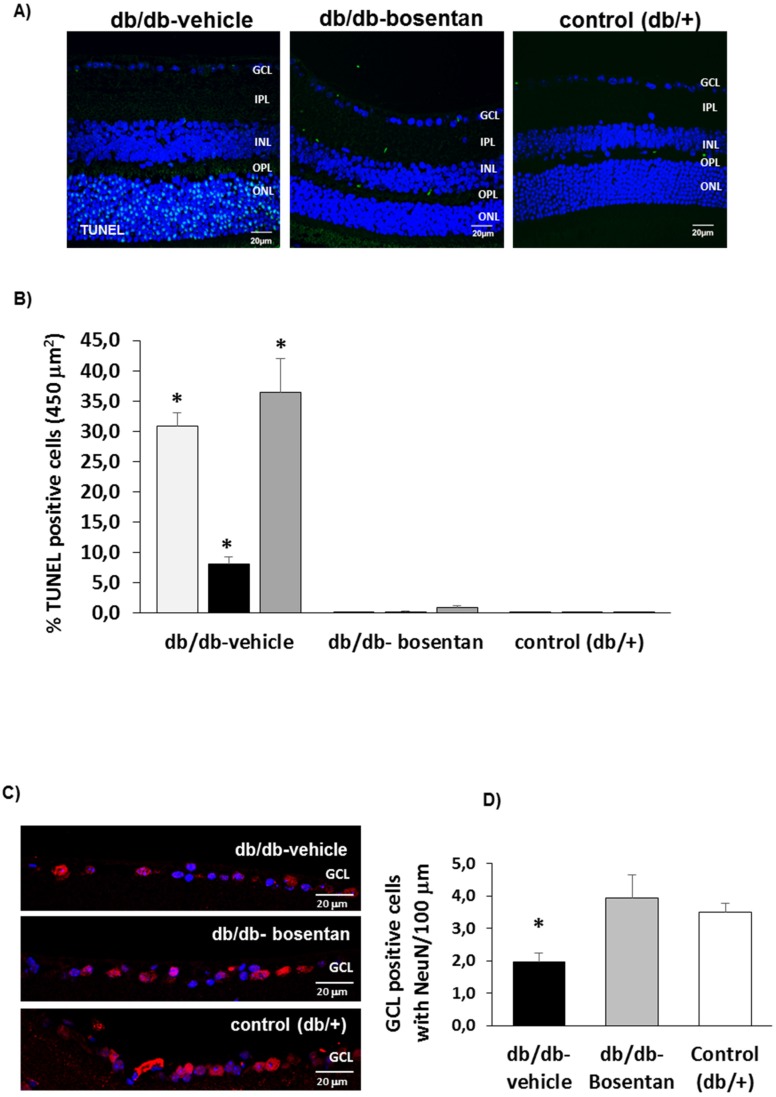
Apoptosis. (**A**) Comparison of TUNEL immunofluorescence (green) between representative samples from a db/db mouse treated with vehicle, a db/db mouse treated with bosentan and a non-diabetic mouse. Scale bar: 20 µm; (**B**) Percentage of TUNEL positive cells in the retinal layers. * *p* < 0.001 in comparison with the other groups in all retinal layers (ONL, INL and GCL). *n =* 6 mice per group (10 sections per retina); (**C**) Comparison of NeuN positive cells (red) between representative samples from a db/db mouse treated with vehicle, a db/db mouse treated with bosentan and a non-diabetic mouse. Nuclei were labeled with DAPI (blue). Scale bar: 20 µm; (**D**) Quantification of NeuN positive cells. * *p* < 0.05 when compared db/db mice treated with vehicle and either db/db mice treated with bosentan and non-diabetic mice.

The apoptosis rate was significantly higher in diabetic mice treated with vehicle than in non-diabetic mice in all retinal layers (Figure 4A,B). Bosentan administration resulted in a significant prevention of apoptosis in all retinal layers (Figure 4A,B). The assessment of retinal ganglion cells in the GCL using NeuN immunofluorescence is showed in Figure 4C,D. The number of ganglion cells was significantly lower in diabetic mice treated with vehicle than in non-diabetic mice. Bosentan administration in diabetic mice significantly prevented the reduction of retinal ganglion cells observed in diabetic mice treated with vehicle.

### 2.6. Bosentan Decreased PKC-β, TNF-α And VEGF Upregulation Induced by Diabetes

#### 2.6.1. In Vivo Studies

**Figure 5:**
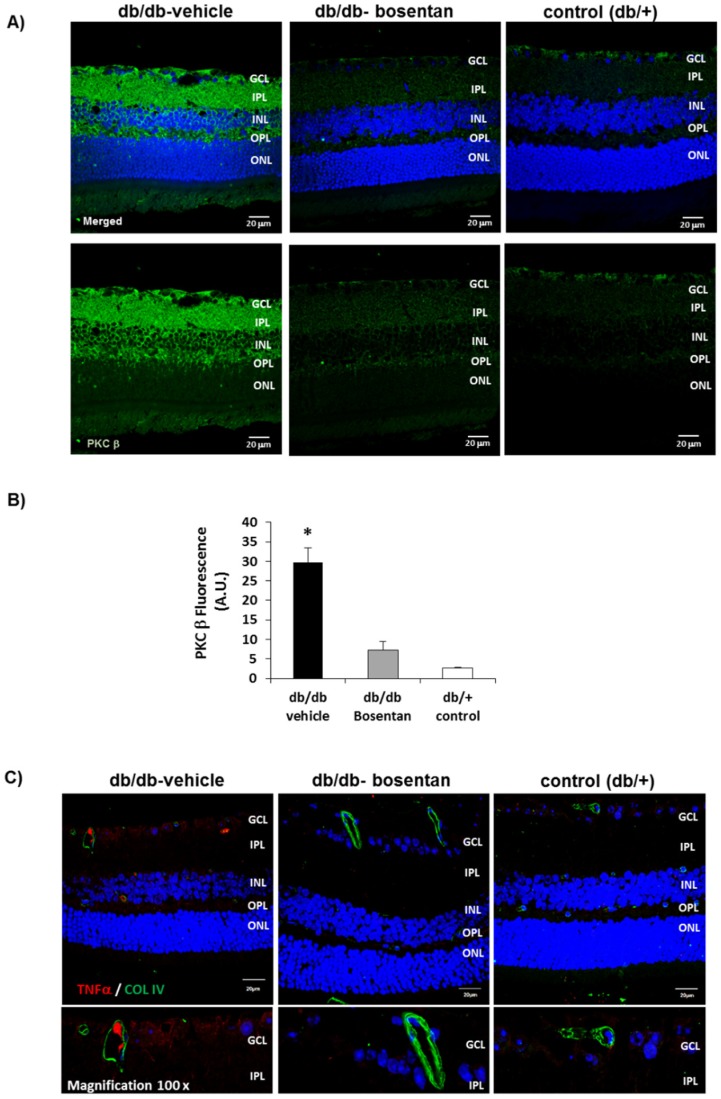
(**A**) Comparison of PKC β immunofluorescence (green) between representative samples from a db/db mouse treated with vehicle, a db/db mouse treated with bosentan, and a non-diabetic mouse (db/+). Nuclei were labeled with Hoechst (blue). Scale bar: 20 µm. GCL: Ganglion cell layer; IPL: Inner plexiform layer; INL: Inner cell layer; OPL: Outer plexiform layer; ONL: Outer nuclear layer; (**B**) Quantification of PKC β immunofluorescence. A.U.: Arbitrary units. Data are expressed as mean ± SD. * *p* < 0.05 in comparison with the other groups; (**C**) TNF-α (red) immunofluorescence retinal images from a representative db/db mouse treated with vehicle, a db/db mouse treated with bosentan and a non-diabetic (db/+) mouse. Blood vessels were immunostained with collagen IV (green). Magnification at 100× shows the expression of TNF-α in retinal vessels in a representative diabetic mouse but not in a representative mouse from the other interventional groups.

We found that in db/db mice aged 12 weeks there was a significant increase of PKC-β in comparison with non-diabetic mice which was prevented by bosentan (Figure 5A,B). Furthermore, we found that bosentan prevents the upregulation of TNF-α induced by diabetes in retinal vessels (Figure 5C).

#### 2.6.2. In Vitro Studies

**Figure 6:**
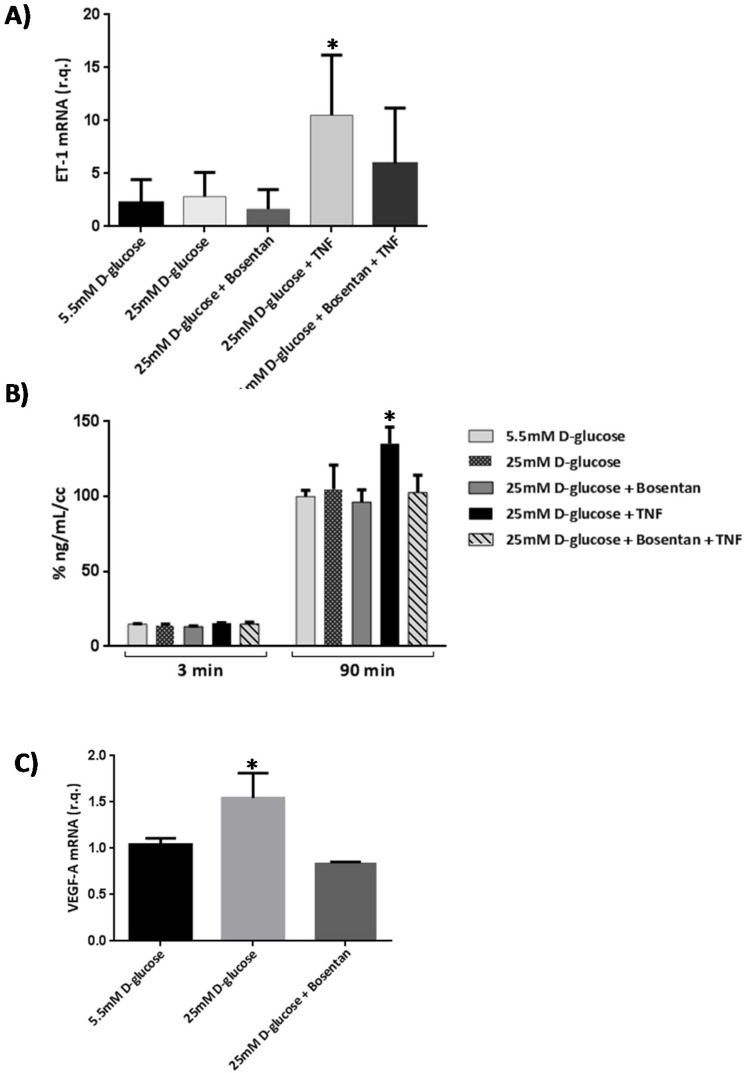
(**A**) ET-1 mRNA expression in human retinal endothelial cells (HRECs) under different treatments including TNF-α (10 ng/mL) and bosentan (30 µM). R.Q.: Relative quantification. Data are expressed as mean ± SD. * *p* < 0.05 in comparison with the other conditions; (**B**) Results of 70 kDa dextran permeability in HRECs in the different conditions examined. The vertical axis is the concentration of dextran. Dextran permeability was measured at 3 and 90 min. * *p* < 0.05 in comparison with the other conditions; (**C**) Effect of bosentan on the upregulation of vascular endothelial growth factor (VEGF) induced by diabetes. * *p* < 0.05 in comparison with the other conditions.

Tumor necrosis factor alpha (TNF-α) induced an increase of mRNA levels of ET-1 in human retinal endothelial cells (HRECs). This upregulation was significantly reduced by bosentan (Figure 6A). The upregulation of ET-1 by TNF-α was associated with a significant increase of permeability that was prevented with bosentan (Figure 6B). Furthermore, bosentan prevented the upregulation of VEGF induced by high glucose in HRECs (Figure 6C).

### 2.7. Pharmacokinetics

**Figure 7:**
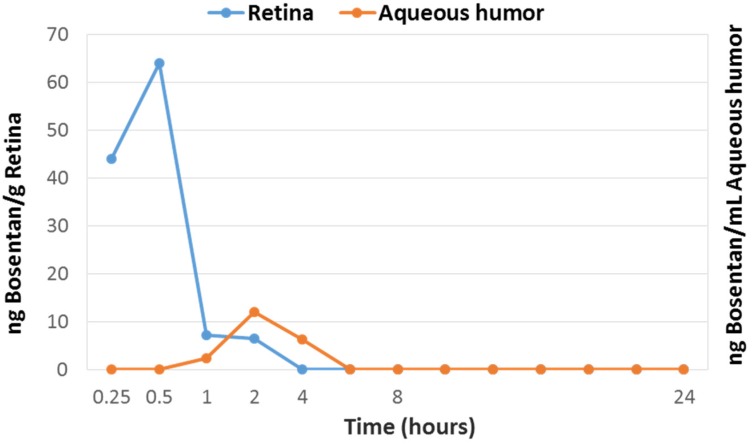
Bosentan content in retina (blue line, ng/g) and aqueous humor (orange line, ng/mL) of rabbits treated with one instillation of bosentan (50 µL, 0.5%).

Bosentan content in ocular tissues and plasma after a single eye drop administration is shown in Figure 7. Bosentan reached the peak level much earlier (0.5 h) before in retina than in the aqueous humor (2 h) and decreased rapidly afterwards. These results suggest that bosentan reaches the back of the eye through the trans-scleral route. Bosentan was detected in plasma only the first 30 min after administration and at very low concentration (0.50 ng/mL at 0.25 h and 0.48 ng/mL at 0.5 h).

## 3. Discussion

Increased circulating levels of ET-1, a potent vasoconstrictor peptide, has been found in patients with diabetes, and a positive association with microangiopathy has been observed [21]. In addition, ET-1 has been reported elevated in the vitreous [22] and aqueous humor [23] of diabetic patients with DR. Moreover, ETA and ETB have been found upregulated in rats with STZ-induced diabetes [17]. In the present study ET-1 and its receptors ETA and ETB were early upregulated in the retina of db/db mice, a model that reproduces type 2 diabetes. In addition, we found for the first time that ET-1 and its receptors are upregulated in human retinas at very early stages of DR. 

The mechanisms involved in the upregulation of ET receptors remain to be elucidated. The dual deleterious effect of ET-1 on both microvasculature and neurons support the concept that ET-1 system plays an essential role in the neurovascular unit impairment [5].

In the present study we provided first evidence that topical ocular treatment with bosentan, a dual endothelin receptor antagonist, resulted in a significant decrease of both glial activation and the rate of apoptosis in comparison with diabetic mice treated with vehicle. The beneficial effect of orally administered ET-1 receptor antagonist on microvascular abnormalities induced by diabetes such as decrease of retinal flow, basement membrane thickening, and capillary degeneration have been previously described [7,8,11,24]. However, to our knowledge, no data regarding the effect of bosentan on retinal neurodegeneration caused by diabetes has been previously reported. Chou et al. [24] reported that the orally administration of atrasentan, a selective ETA receptor antagonist, was able to ameliorate vascular regression in db/db mouse. However, the effect on neuroretinal apoptosis measured by TUNEL was only partial. This finding supports the idea that the blockage of the ETB receptor is essential to prevent retinal neurodegeneration induced by diabetes. In support of this notion it has been reported that ETB receptors are involved in retinal ganglion cell loss induced by glaucoma [13,14] and by optic nerve injury [15]. 

ET-1 expression induced by hyperglycemia in diabetes is partly due to activation of PKC-beta and -delta isoforms [25]. It should be noted that the PKC β isoform is involved in diabetic complications and in particular in DR. In fact, it has been considered a therapeutic target [26]. Notably, in the present study we found that bosentan prevented the upregulation of PKC β induced by diabetes in the neuroretina.

In addition, we found that bosentan inhibited the upregulation of ET-1 induced by TNF-α in HRECs. Moreover, bosentan significantly reduced the hyperpermeability induced by TNF-α in HRECs. Furthermore, bosentan topically administered was able to prevent the upregulation of TNF-α in retinas from diabetic mice. It should be noted that TNF-α participates in the breakdown of the blood-retinal barrier (BRB) by downregulating tight junction proteins of endothelial cells and favoring VEGF-induced permeability [27]. Therefore, bosentan by inhibiting the upregulation of both TNF- α and VEGF would also play a significant role in preventing the hyperpermeability induced by diabetes.

The results of the pharmacokinetic study performed on in rabbits suggest that bosentan is mainly reaching the retina through the trans-scleral route. In addition, the pass to systemic circulation is negligible.

In conclusion, we found that upregulation of ET-1 and its receptors (ETA and ETB) is an early event in the diabetic retina, the topical (eye drops) administration of bosentan prevents retinal neurodegeneration induced by diabetes not only by blocking but also by downregulating ETB receptors. The inhibition of the diabetes-induced upregulation of PKC-β, TNF-α and VEGF induced by diabetes seems to play an important role in the beneficial vascular action of bosentan.

## 4. Materials Material and Methods

### 4.1. Human Retinas

Human postmortem eye cups were obtained from non-diabetic (*n =* 12) and diabetic eye donors (*n =* 12) free of funduscopic abnormalities or with mild DR in the ophthalmological examinations performed during the preceding 2 years, matched by age (68 ± 8 vs. 69 ± 7 years), and selected from our Eye Bank (Vall d′Hebron Research Institute, Barcelona, Spain). The procedure for eye cup donation and for the handling of this material was regulated by the protocol of donations of the Blood and Tissue Bank of the Catalan Department of Health and was approved by the ethics committee (CEIC, November, 2011). 

Reverse transcription polymerase chain reaction were carried out from the cDNA of each condition using SYBR Green PCR Master Mix (Applied Biosystems, Warrington, UK) using the 7900 HT Sequence Detection System in 384-well optical plates with specific primers for ET-1 (forward: 5′-TCGTCCCTGATGGATAAAGA-3′; reverse: 5′-GGCAAAAATTCCAGCACTTC-3′), hETA (forward: 5′-AAGGAATGGGAGCTTGAGAA-3′; reverse 5′-CAGAGGCATGACTGGAACA-3′), hETB (forward: 5′-TTTGCCTGGTCCTTGTCTTT-3′; reverse: 5′-AAGCACGACTGCTTTTCCTC-3′). For each sample, qPCR was performed in duplicate and relative quantities were calculated using ABI SDS 2.0 RQ software (Applied Biosystems, Madrid, Spain) and the 2^−ΔΔ*C*t^ analysis method with human β-actin as the endogenous control (forward: 5′-AGGCCAACCGCGAGAAGATGACC-3′; reverse 5′-GAAGTCCAGGGCGACGTAGCAC-3′). Each sample was assayed in duplicate, and negative controls were included in each experiment.

### 4.2. Animals

The neuroprotective effect of eye-drops containing bosentan was tested in the db/db mouse model. This mouse carries a mutation in the leptin receptor gene and is a model for obesity-induced type 2 diabetes. A total of 12 male db/db (BKS.Cg- + Lepr db/+ Lepr db/OlaHsd) mice aged 10 weeks were purchased from Charles River Laboratories. In addition, 6 non-diabetic (db/+) mice matched by age were used as the control group.

Bosentan or vehicle eye drops were administered directly onto the superior corneal surface of each eye using a syringe. One drop (5 µL) of bosentan (0.5%) or vehicle was administered twice daily for 15 days. On day 15, mice were euthanized by cervical dislocation and the eyes were immediately enucleated.

The pharmacokinetic profile of bosentan was studied in 35 pigmented HY79b female rabbits.

This study was approved by the Animal Care and Use Committee of VHIR (Vall d’Hebron Research Institute, CEEA 75/15, September 2015). All the experiments were performed in accordance with the tenets of the European Community (86/609/CEE) and ARVO (Association for Research in Vision and Ophthalmology).

### 4.3. Immunohistochemistry for Endothelin (ET-1) And Endothelin Receptors (ETA-R and ETB-R)

All eyes were fixed in 4% paraformaldehyde after enucleation. Immunohistochemical studies were done on 4 µm-thick paraffined sections of the eyes thaw-mounted onto poly-l-Lysine treated slides and heated in an oven at 65 °C for 1 h to promote adherence to the slide. These sections were fixed in methanol/acid, rehydrated and washed in 0.01-M phosphate buffered saline (PBS) and incubated in blocker (5% BSA, and 5% goat serum in PBS) for 1 h at room temperature. Then, they were incubated overnight at 4 °C with a rabbit anti-ET-1 (Ab 117757) diluted 1:1000 (prepared in 1% BSA in PBS); rabbit anti-ETA (Ab 117521) (Abcam Ltd., Cambridge, UK) and rabbit anti-ET-B (Millipore 3284) at 1:500 dilution (prepared in 1% BSA in PBS). After three washes in PBS, the sections were incubated with secondary antibody Alexa Fluor 594 conjugate for ET-1 or Alexa Fluor 488 conjugate for ETA and ETB (Molecular Probes-Life Technologies, Grand Island, USA). The sections were washed three times in PBS, counterstained with Hoestch and mounted with Mounting Medium Fluorescence (Prolong, Invitrogen, PO BOX 6482, Carlsbad CA, 92008 USA) with a coverslip.

The immunofluorescence was quantified using laser confocal microscopy (Fluoview FV 1000 Laser Scanning Confocal Microscope Olympus; Olympus, Hamburg, Germany) and ImageJ software (ImageJ Software with 64-bit Java 1.8.0_112, Version 2018, National Institutes of Health, Bethesda, MD, EEUU).

### 4.4. Neurodegeneration Measurements

#### 4.4.1. Measurements of Glial Activation

Glial activation was evaluated by Laser Scanning Confocal microscopy using specific antibodies against GFAP. Sections were fixed in acid methanol (−20 °C) for 2 min, followed by three washes with PBS. Sections were permeabilized with TBS-Triton X-100 0.025% and were incubated in blocker (1% BSA, and 10% goat serum in PBS) for 2 h at room temperature. Sections were then incubated with rabbit anti-GFAP (Abcam Ltd., Cambridge, UK) (1:500 dilution prepared in blocking solution) overnight at 4 °C in a humid atmosphere. After three washes in PBS, the sections were incubated with secondary antibody Alexa 488 goat-anti-rabbit (Invitrogen) (1:200 dilution prepared in blocking solution). The sections were washed three times in PBS, counterstained with Hoestch and mounted with Mounting Medium Fluorescence (Prolong, Invitrogen) and mounted with a coverslip. Comparative digital images from samples were recorded with a Fluoview FV 1000 Laser Scanning Confocal Microscope Olympus using identical brightness and contrast settings.

To evaluate the degree of glial activation, we used a scoring system based on the extent of GFAP staining previously described [28]. This scoring system was as follows: Müller cell endfeet region/ganglion cell layer (GCL) only (score 1); Müller cell endfeet region/GCL plus a few proximal processes (score 2); Müller cell endfeet plus many processes, but not extending to Inner nuclear layer (INL) (score 3); Müller cell endfeet plus processes throughout with some in the ONL (score 4); Müller cell endfeet plus lots of dark processes from GCL to outer margin of ONL (score 5).

#### 4.4.2. Immunohistochemical Analysis for Apoptosis Assessment

The TUNEL (Terminal Transferase dUTP Nick-End Labeling) staining was carried out using the DeadEnd Fluorometric TUNEL System kit (PROMEGA, Madison, WI, USA). Cryosections of retina were permeabilized by incubation for 2 min on ice with 0.1% Triton X-100 in 0.1% sodium citrate, freshly prepared. The secondary antibody was Alexa 488 goat-anti-rabbit (Invitrogen, San Diego, CA, USA). For evaluation by Laser Scanning Confocal microscopy the excitation wavelength was 488 nm and detection in the range of 515–565 nm (green) was used.

In order to clearly differentiate the ganglion cells from astrocytes within the GCL, an immunostaining for neuronal-specific nuclear protein (NeuN) was performed. Neuronal-specific nuclear protein was evaluated by immunofluorescence using a specific monoclonal antibody (anti-NeuN; ab104224; Abcam; Cambridge, UK).

#### 4.4.3. Other Immunohistochemical Analysis

We analyzed the PKC β protein, an important mediator of diabetic complications, which regulates ET-1 in the retinal vascular cells (ab189782; Abcam; Cambridge, UK). In addition, we evaluated the protein expression of TNF-α (ab8348; Abcam). Blood vessels were immunostained with collagen IV (ab6586; Abcam). For all the immunofluorescence analyses, after incubation with the secondary antibodies, the sections were counterstained with Hoetchst and mounted with Mounting Medium Fluorescence (Prolong, Invitrogen) with a coverslip. Comparative digital images from samples were recorded with a Fluoview FV 1000 Laser Scanning Confocal Microscope Olympus using identical brightness and contrast settings.

#### 4.4.4. Pharmacokinetic Analyses

A pharmacokinetic analysis after a single topical instillation of bosentan (0.5%) was performed. For this purpose, a single instillation in the right eye was administered to 35 female pigmented HY79b rabbits that were randomly distributed as summarized in Table 1. Bosentan concentration in plasma, aqueous humor and retina was measured using HPLC with MS/MS detection. This method was validated with respect to selectivity, linearity, precision, accuracy, matrix effect, extraction efficiency and dilution integrity. The pharmacokinetic parameters calculated included maximum observed concentration and time to maximum concentration.

### 4.5. In Vitro Studies in Human Retinal Endothelial Cells

Human retinal endothelial cell (HREC) cultures were obtained from a vial of cryopreserved cells purchased from Innoprot (Biscay, Spain). Human retinal endothelial cells were thawed in the laboratory and cultured in endothelial basal medium (EBM) containing 5.5 mM d-glucose, 5% FCS, 100 U/mL penicillin, and 100 µg/mL streptomycin and ECGS supplement (Innoprot, Biscay, Spain). Human fibronectin (Merck Millipore, Madrid, Spain) was used at 5 µg/mL for cell attachment. The endothelial medium was changed every 3 days. Human retinal endothelial cells from passage 3 were used for the experiments and grown up to confluence. For cell stimulation, culture media were changed to a medium supplemented with 1% FBS. Cells were treated with TNF-α (10 ng/mL) (Miltenyi, Madrid, Spain) and bosentan (30 µM) for the final 24 h of the experiment.

#### 4.5.1. Measurement of HREC Permeability

Permeability in HREC monolayers was obtained on permeable supports at 1.2 × 10^5^ cells/well, PCF filters (MerkMillipore, Madrid, Spain). Inserts were incubated for 48 h at 37 °C in 5% CO_2_-air to form the monolayer. Then, the medium was serum depleted on the upper side for 14 h before the treatments. Bosentan in PBS (30 µM) was include 1 h before TNF-α. Changes in the permeability were detected by changes in the fluorescence on the basal side of the insert. A total of 100 µg/mL of fluorescent FITC-Dextran (70 KDa) (Sigma, Madrid, Spain) was added to the upper side of the insert. Aliquots of 200 µL from the basal compartment were read in a SpectraMax Gemini (Molecular Devices, Sunnyvale, CA, USA). Dextran data was obtained by extrapolation in a standard curve. Each condition was tested 3 times.

#### 4.5.2. RNA Extraction and Quantitative Real-Time PCR

Human retinal endothelial cell RNA was extracted with Nzyol (Nzytech, Lisbon, Portugal). Reverse transcription polymerase chain reaction were carried out using SYBR Green PCR Master Mix (Applied Biosystems, Warrington, UK) using the 7900 HT Sequence Detection System in 384-well optical plates with specific primers for human VEGF: 5′-TGCATTCACATTTGTTGTGCTGTAG-3′ and 5′-GCAGATTATGCGGATCAAACC-3′. The primers for human ET-1 and human β-actin are detailed above. For each sample, qPCRs were performed in triplicate and relative quantities were calculated using ABI SDS 2.0 RQ software and the 2^−∆∆*C*t^ analysis method with human β-actin as the endogenous control.

### 4.6. Statistical Analysis

Data are presented as mean ± SD. Comparisons of continuous variables were performed using ANOVA and Student’s *t* test. Comparisons of categorical variables were performed using the Fisher test. Levels of statistical significance were set at *p* < 0.05.

## Figures and Tables

**Table 1:** Design of the pharmacokinetic study.

Groups	Dose Regimen	Time-Points Post Dose	Animal Number
1	Single 50 µL instillation in right eye	0.25 h	5
2		0.5 h	5
3		1 h	5
4		2 h	5
5		4 h	5
6		8 h	5
7		24 h	5
